# Beriberi: A Reversible Cause of Acute Severe Pulmonary Hypertension

**DOI:** 10.7759/cureus.27376

**Published:** 2022-07-27

**Authors:** Mei L Tan, Christopher G Willis

**Affiliations:** 1 General Medicine, Khoo Teck Puat Hospital, Singapore, SGP

**Keywords:** beriberi, pulmonary hypertension, high output heart failure, thiamine, lactate, cardiomegaly

## Abstract

Thiamine, also known as vitamin B1, plays a fundamental role in energy metabolism. The organs most sensitive to thiamine deficiency are the cardiovascular and nervous systems. The usual presentations include Wernicke's encephalopathy, polyneuropathy (known as "dry beriberi"), and the cardiovascular form (known as "wet beriberi"). Wet beriberi-induced acute severe pulmonary hypertension has rarely been previously described. Here we present a case of wet beriberi with severe right heart failure due to acute pulmonary hypertension. The underlying physiologic derangements dramatically improved after thiamine treatment. No other possible causes of pulmonary hypertension could be identified, with the exception of thiamine deficiency. This case illustrates the importance of considering wet beriberi as a possible cause of acute pulmonary hypertension with right heart failure.

## Introduction

Thiamine deficiency is probably much more common than currently recognized in developed countries, including in our local context in Singapore. The presentation can be that of Wernicke's encephalopathy, "dry beriberi", "wet beriberi", and Shoshin beriberi (sho = acute damage, shin = heart) associated with shock, or even a combination of the above. Cardiovascular beriberi has a number of variant presentations that doctors need to be familiar with. Early diagnosis and treatment with intravenous thiamine are the keys to preventing a bad outcome.

Some history is in order. Major epidemics really started to occur around the world from about 1870, even though symptoms compatible with beriberi were described centuries ago. It was the era of Western European colonizing, and, all around the world in the colonies, thousands of beriberi cases were occurring. So many cases, in fact, that specialized beriberi hospitals were built. These were often situated at the seaside as one of the early theories was that the disease was due to bad air, known as "miasmus". Singapore had one such hospital from 1907-1925 [1]. Numerous research teams sought to determine the underlying cause of the condition, and there was much dispute. Although many researchers early on suspected a dietary deficiency as causative, it took nearly 50 years before the responsible molecule, thiamine, was isolated. It came to be referred to as vitamin B1 [2].

Why was 1870 so special? It so happened that was the year a new technology for processing grains began to be used. The impetus for this change was the fact that the shelf life of grains was relatively short, a matter of weeks. Grains quickly went rancid due to the presence of surface-layer oils. If the pericarp layer was removed, the grains would keep very much longer. Removal of the pericarp solved one problem but unknowingly created another because most of the vitamins in grains are found in the "germ", which is situated in the pericarp [2].

With the discovery and synthesis of thiamine, governments around the world mandated the fortification of milled grains with thiamine as part of public health policy [2]. Consequently, beriberi became a rare condition, unfamiliar to most doctors. However, over many decades, widespread lapses in thiamine fortification have started to occur [3].

Thiamine is a small water-soluble molecule with a hexa- and penta ring. It is excreted in the urine. Daily thiamine requirements are proportional to carbohydrate intake, with roughly 0.4mg required per 1000 kcal ingested. Under normal circumstances, men need about 1.2mg/day and women 1.0mg/day. Body stores are limited and can be used up in a matter of weeks when the diet lacks thiamine. There are ways that thiamine deficiency can occur besides simple dietary lack. Urinary losses increase in those taking diuretics [4]. Over-cooking of food destroys thiamine. Tea and coffee are anti-thiamines. Thiaminases contained in raw fish, shellfish, and beetle nuts may cleave the thiamine molecule. Alcohol causes jejunal thiamine malabsorption, as may also occur following bariatric surgery, in chronic diarrhea, or in other malabsorption syndromes. High carbohydrate intake causes greater thiamine utilization, as does pregnancy and lactation, thyrotoxicosis and sepsis, and other chronic inflammatory states [5].

## Case presentation

**Figure 1:**
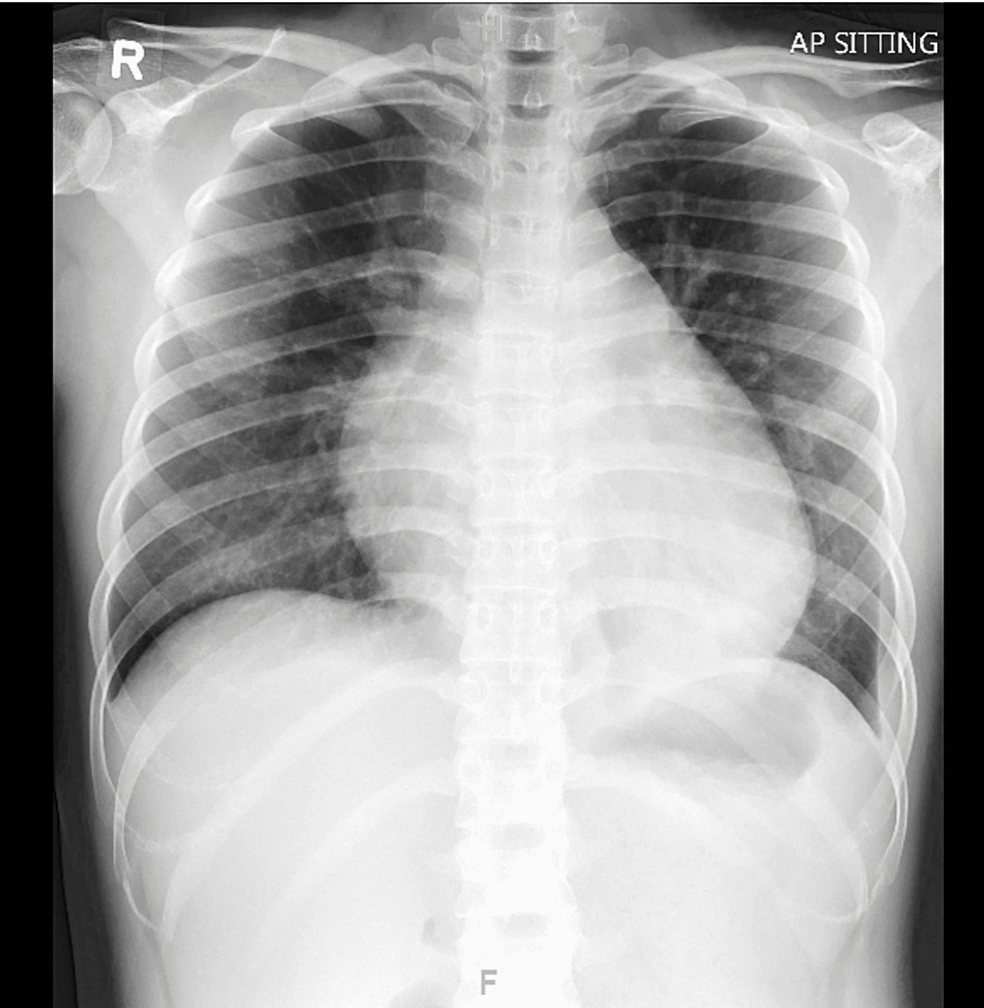
Chest X-ray showing that the heart is moderately enlarged and globular with prominent vascular markings

A 23-year-old Myanmar female domestic worker presented to Khoo Teck Puat Hospital with palpitations, acute dyspnea, and lower limb edema. This had developed rapidly over a week following a brief viral illness which had resulted in anorexia and vomiting. Clinical examination revealed an ill-looking and anxious young woman with a mild fever of 38.6 degrees Celsius, a rapid pulse of 113 beats/min, and a respiratory rate of 24 breaths/min. She was initially normotensive at 104/82 mmHg but soon had a significant decline in diastolic pressure to around 40 mmHg. Clinical examination revealed a very raised jugular venous pulse with obvious giant 'V' waves. There was a marked left parasternal heave suggestive of severe right heart strain and dilatation. The apical impulse was not palpable. The second heart sound S2 was persistently widely split, and a right-sided third heart sound S3 was present. There was moderate pitting edema of both legs below the mid-shin. Oxygen saturation was normal, as were the breath sounds. There was mild epigastric tenderness but no rebound or guarding. Laboratory tests reported normal hemoglobin with mild neutrophilia, normal urea and creatinine, elevated lactate level at 3.5mmol/L with mild high anion gap metabolic acidosis, mild transaminase elevations, mild troponin elevation, and normal thyroid function tests. A chest X-ray showed significant cardiomegaly without pulmonary edema (Figure 1).

**Figure 2:**
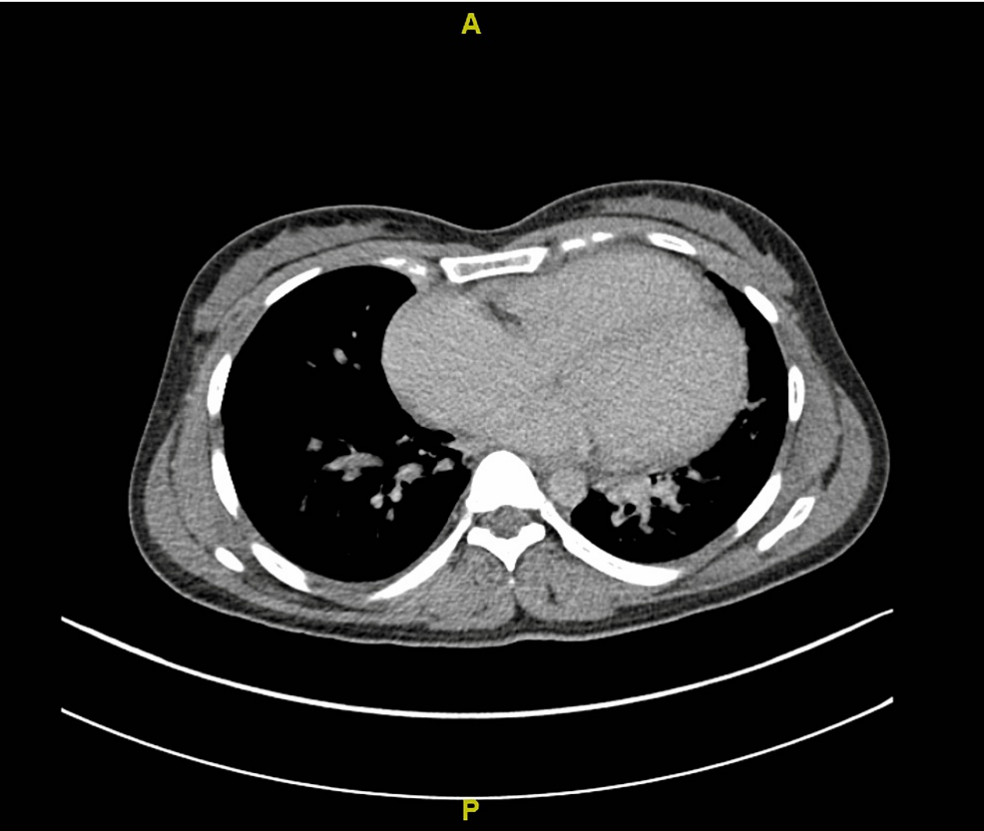
Contrast CT chest showing four-chamber dilatation of the heart with no pulmonary embolism

Electrocardiogram demonstrated sinus tachycardia with right axis deviation. The clinical impression of acute severe pulmonary hypertension was confirmed by an urgent echocardiogram which showed a left ventricular ejection fraction (LVEF) of 50% with dilated right heart chambers and severe right heart strain. She was given intravenous antibiotics for the empiric cover of possible bacterial infection and prophylaxis cover for venous thrombosis with subcutaneous low molecular weight heparin. Based on the physician's experience and background in managing patients with thiamine deficiency, intravenous thiamine 500mg every eight hours was prescribed. An urgent CT-pulmonary angiogram revealed no evidence of pulmonary embolism (Figure 2).

**Figure 3:**
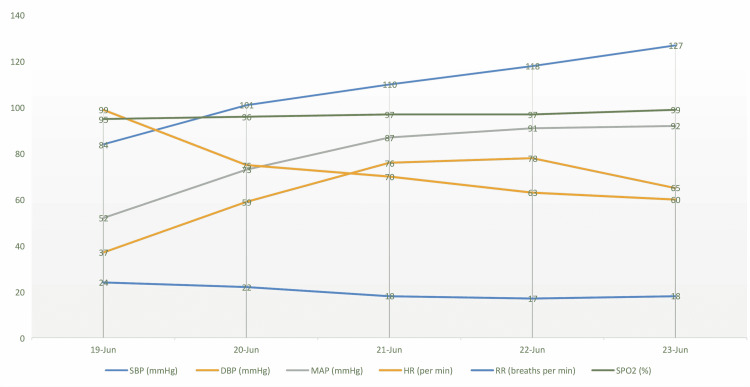
Physiological changes over time shown in a graph after the administration of intravenous thiamine with an immediate reduction in pulse rate, improvement of blood pressure, and reduction in respiratory rate SPB - systolic blood pressure; DBP - diastolic blood pressure; MAP - mean arterial pressure; HR - heart rate; RR - respiratory rate; SPO2 - oxygen saturation

Within hours of receiving thiamine, her vital signs were steadily improving, as demonstrated in Figure 3. It was then discovered that she had also developed numbness and weakness of both legs in an ascending pattern. Her cardiovascular presentation of "wet" beriberi recovered quickly, whereas her peripheral neuropathy recovered over several weeks. Further history-taking revealed that her diet consisted mainly of polished white rice and home-grown vegetables with no meat included in her main meals.

## Discussion

Beriberi is often considered a disease of the past, and very few doctors are familiar with its presentation. Cardiovascular or "wet" beriberi has various presentations attributable to the fact that thiamine deficiency causes important physiologic derangements, particularly capillary leak and low systemic vascular resistance due to peripheral vasodilatation. Capillary leak is the most common and results in peripheral edema and, when severe, may include third-spacing into the pleural and peritoneal spaces. The lowering of systemic vascular resistance tends to be more subtle, but as it worsens, it results in low diastolic pressure, tachycardia, and hyper-dynamic circulation. Typically, an echocardiogram will show normal cardiac function. However, another physiologic derangement can occur rarely, namely the mitochondrial failure of energy production, with resultant lysis of the cardiac myocytes, and this can result in severe left ventricular failure. This presentation is known as "Shoshin beriberi". Another rare and possibly under-recognized presentation is that of acute pulmonary hypertension as occurred in our case. The literature has a smattering of case reports on this [6-12]. Prompt diagnosis with the administration of intravenous thiamine is lifesaving. In critically ill patients, the presence of peripheral neuropathy is usually discovered after the initial cardiovascular crisis has settled. 

In most cases, the diagnosis of thiamine deficiency in its varied presentations has been made on clinical grounds together with taking a dietary history rather than by any blood or urine test. In fact, very few laboratories offer thiamine testing. If such testing is done, it is usually a send-out test, and the results take time to be reported. For the very few laboratories that do offer testing, the main tests are usually serum or urine thiamine and, less often, a red blood cell or erythrocyte transketolase activity (ETKA). None of these tests are considered ideal. A person may have very low thiamine tissue stores, and yet the serum and urine thiamine levels may be normal if the person happened to have just eaten a meal containing a significant amount of thiamine. The ETKA test is a better reflection of thiamine stores, but it is still not robust because only a tiny percentage of thiamine is stored in the erythrocytes [2].

The major sources of thiamine can be found in whole grains, cereals, nuts, seeds, meat (especially pork, with much less in fish, poultry, beef, and lamb), round beans, peas, lentils, soy and Marmite, Vegemite, and Bovril. An easy way to remember this list is to think in terms of items that consist of a kernel, either nuts or seeds, as this covers most of this list. It is important to ask the right questions in taking a dietary history. Start by asking if the person takes any vitamins, and if so, what type. Do they eat white rice or brown rice? Do they eat bread? If so, what type or brand (not all breads are made with thiamine-fortified flour, so you need to familiarize yourself with what the situation in your region is like). Do they eat nuts (peanuts, cashews, almonds, pistachios, macadamia) or seeds (chia, flax, sunflower, sesame)? Do they eat beans, peas, lentils, or soybeans? If they eat meat, what type of meat and how do they cook it? As you go through this list with the patient, you need to get a rough idea of how much and how often they eat these particular items. Refined rice, sugar, and several processed foods are low in vitamin B1, as are most fruits and vegetables. As described in this case presented, this foreign domestic worker was eating a high-energy diet, which mainly contained white rice. This would increase her daily thiamine requirements and thus result in deficiency unless a thiamine supplement was available [13].

With high clinical suspicion, such cases can easily be treated with appropriate replacement of thiamine and, to a large extent, prevent devastating consequences and significantly improve cardiac function. The diagnosis of this disease depends on the following three factors: 1) clinical symptoms suggestive of "heart failure" and a characteristic history of dietary inadequacy; 2) exclusion of other etiologies of heart disease; and 3) therapeutic response to thiamine administration.

Knowing that waiting for the measurement of thiamine will result in a delay in diagnosis, treatment with thiamine should be instituted immediately. If the patient's response to this empirical thiamine replacement is favorable, it is reasonable to conclude that the "heart failure" is attributable to thiamine deficiency. This approach is safe as thiamine is non-toxic, even at high blood levels.

## Conclusions

In conclusion, the diagnosis of beriberi requires a high index of suspicion owing to the non-specific symptoms and signs. It is essential to be familiar with the various presentations that can occur, including those which are little-known. In this case, the unusual presentation was acute life-threatening pulmonary hypertension with right heart failure. Failure to give thiamine may have resulted in death. In fact, it is good practice to give thiamine supplementation for all cases of cardiac failure. Although this condition is less commonly seen in Singapore, clinical awareness has to be reinforced due to the dynamic influx of foreign workers from neighboring countries. At the same time, public health efforts to eliminate thiamine deficiency are the key to prevention by promoting brown rice and fortifying flour with thiamine.
